# Quit and Smoking Reduction Rates in Vape Shop Consumers: A Prospective 12-Month Survey

**DOI:** 10.3390/ijerph120403428

**Published:** 2015-03-24

**Authors:** Riccardo Polosa, Pasquale Caponnetto, Fabio Cibella, Jacques Le-Houezec

**Affiliations:** 1 Centro per la Prevenzione e Cura del Tabagismo (CPCT), Azienda Ospedaliero-Universitaria “Policlinico-Vittorio Emanuele”, Università di Catania, Catania 95100, Italy; E-Mail: p.caponnetto@unict.it; 2 Dipartimento di Medicina Clinica e Sperimentale, Università di Catania, Catania 95100, Italy; 3 National Research Council of Italy, Institute of Biomedicine and Molecular Immunology, Palermo 90100, Italy; E-Mail: fabio.cibella@ibim.cnr.it; 4 Addiction Research Unit at INSERM 1178 (Mental and Public Health), 75014 Paris, France; E-Mail: jacques.lehouezec@amzer-glas.com; 5 UK Centre for Tobacco Control Studies, University of Nottingham, Nottingham NG51PB, UK

**Keywords:** smoking cessation, smoking reduction, electronic cigarette, vape shop, tobacco harm reduction

## Abstract

*Aims*: Here, we present results from a prospective pilot study that was aimed at surveying changes in daily cigarette consumption in smokers making their first purchase at vape shops. Modifications in products purchase were also noted. *Design*: Participants were instructed how to charge, fill, activate and use their e-cigarettes (e-cigs). Participants were encouraged to use these products in the anticipation of reducing the number of cig/day smoked. *Settings*: Staff from LIAF contacted 10 vape shops in the province of the city of Catania (Italy) that acted as sponsors to the 2013 No Tobacco Day. *Participants*: 71 adult smokers (≥18 years old) making their first purchase at local participating vape shops were asked by professional retail staff to complete a form. *Measurements*: Their cigarette consumption was followed-up prospectively at 6 and 12 months. Details of products purchase (*i.e.*, e-cigs hardware, e-liquid nicotine strengths and flavours) were also noted. *Findings*: Retention rate was elevated, with 69% of participants attending their final follow-up visit. At 12 month, 40.8% subjects could be classified as quitters, 25.4% as reducers and 33.8% as failures. Switching from standard refillables (initial choice) to more advanced devices (MODs) was observed in this study (from 8.5% at baseline to 18.4% at 12 month) as well as a trend in decreasing the e-liquid nicotine strength, with more participants adopting low nicotine strength (from 49.3% at baseline to 57.1% at 12 month). *Conclusions*: We have found that smokers purchasing e-cigarettes from vape shops with professional advice and support can achieve high success rates.

## 1. Introduction

Most smokers want to quit and make attempts to do so, but the majority of these attempts fail largely because the powerful addictive qualities of nicotine and non-nicotine sensory and behavioural cues [1,2]. For those willing to quit, combination of pharmacotherapy and intensive behavioural intervention for smoking cessation can support their quit attempts and can double or triple quit rates [3,4]. However, outside the context of rigorous randomized controlled trials, reported efficacy rates are somewhat lower [5,6,7]. Consequently, the need for novel and more efficient approaches to smoking cessation interventions is unquestionable.

Electronic cigarettes (e-cigs) are an attractive long-term alternative nicotine source to conventional cigarettes because of their many similarities with smoking [8,9] and randomized controlled trials with early generation products have shown that they may assist smokers to remain abstinent during their quit attempt [10,11]. E-cigs come in all sorts of shapes and sizes. Some, commonly referred to as first generation devices, resemble tobacco cigarettes (cigalikes) with a mouthpiece resembling a cigarette filter, a battery and a LED which glows when the user inhales on the device. These devices comprise low-capacity disposable or re-chargeable batteries and combined cartridges and atomisers (cartomisers). Second generation devices often resemble a pen (personal vaporizer) are equipped with high-capacity lithium batteries, a more efficient vaporizing system compared to cigalikes and can be refilled with a wide combination of flavours and nicotine levels. These devices assent to a more fulfilling vaping experience compared to first generation e-cigs with the choice of an extensive number of e-liquid aromas, and thicker vapour [12,13].

Third generation devices (more advanced devices-MODs) bear little visual resemblance to cigarettes, use larger-capacity batteries, replacement heating coils and wicks for atomizers, and adjustable and programmable power delivery.

These products can be purchased in tobacco retail environments, convenience stores, liquor stores, pharmacies, and on the Internet. Shops devoted exclusively to trial and sales of e-vapour products (e.g., refillable and disposable e-cigs, several types of solution strengths and flavours, customizable atomizers and tank systems, and other accessories) are known as “vape shops” and their popularity has been growing in parallel to that of e-cigs [14].

Two randomised controlled trials investigating success rates in smokers asked to try cigalikes have reported disappointingly low quit rates; 4%–8.7% for the ECLAT study in Italy [10] and 4%–7.3% for the ASCEND study in New Zealand [11]. Not surprisingly, much higher success rates have been reported in clinical trials with refillable penlike e-cigs, with an overall quit rate of 36% at 6 months [15,16]. Nonetheless, it is likely that their performance and appeal as cigarette substitutes can be further improved outside the rigid context of an experimental setting by describing success rates with refillables purchased by smokers at vape shops where professional advice and regular technical support it is also available. Therefore, we hypothesized that vape shops environment together with best matched e-vapour products may promote high success rates in smokers interested in trying this alternative to tobacco smoking. Here, we present results from a prospective pilot study that was aimed at surveying changes in daily cigarette consumption in smokers making their first purchase at vape shops. Modifications in products purchase over time were also noted.

## 2. Methods

### 2.1. Participants and Study Design

Adult smokers (≥18 years old) making their first purchase at local participating vape shops were asked by professional retail staff to complete a form with their basic demographic and smoking history details together with scoring of their level of nicotine dependence by means of Fagerstrom Test of Nicotine Dependence (FTND) questionnaire [17]. Participants were instructed how to charge, fill, activate and use their e-cigs. Key troubleshooting was addressed and phone numbers were supplied for technical assistance. Participants were encouraged to use these products in the anticipation of reducing the number of cig/day smoked. Their cigarette consumption was followed-up prospectively at 6 and 12 months. Details of products purchase (*i.e.*, e-cig hardware, e-liquid nicotine strengths and flavours) were also noted. University of Catania Ethics Review Board approved the study protocol and subjects gave consent prior to participation.

### 2.2. Vape Shops

Staff from Lega Italiana Anti Fumo (LIAF) contacted 10 vape shops in the province of the city of Catania (Sicily) that acted as sponsors to the 2013 No Tobacco Day. Vape shop owners were asked to help with a survey of smokers making their first purchase at their vape shops. Three declined, but seven accepted to be involved. Participating shops were bar or lounge types and displayed a wide range of nicotine in juices, large selection of flavours and hardware (including cigalikes, refillables and MODs).

### 2.3. Study Outcome Measures

Sustained 50% reduction in the number of cig/day from baseline (*reducers*) was defined as sustained self-reported 50% reduction in the number of cig/day compared to baseline for the 30-day period prior to follow-up visit.

Sustained 80% reduction in the number of cig/day (*heavy reducers*) and sustained smoking abstinence from baseline (*quitters*) were defined as sustained self-reported 80% reduction in the number of cig/day compared to baseline and complete self-reported abstinence from tobacco smoking (not even a puff) for the 30-day period prior to follow-up visit respectively. Smokers who failed to meet the above criteria and those who were lost to follow-up were categorized as reduction/cessation failures (*failures*).

### 2.4. Statistical Analyses

Primary and secondary outcome measures were computed by including all enrolled participants and assuming that all those individuals who were lost to follow-up are classified as failures (intention-to-treat analysis). Data were expressed as mean (±SD). One-way Analysis of Variance (ANOVA) was used for detecting differences between means, and χ^2^ test for testing differences in variable frequency distributions. Repeated Measures ANOVA was used for detecting differences at different time points.

## 3. Results

### 3.1. Participant Characteristics

A total of 71 (M 44; F 27) regular smokers (mean [±SD] pack/years of 32.4 [±13.7]) with a mean (±SD) age of 41.7 (±8.8) years, and mean (±SD) FTND score of 5.6 (±2.2) were enrolled by seven participating vape shops (Table 1). Retention rate was high, with 49 (69%) participants completing all study visits and attending their final follow-up visit at 12 month. Baseline characteristics (sex, age, pack/year, and FTND) of those who were lost to follow-up were not significantly different from those of participants who completed the study.

**Table 1:** Characteristics of the study sample at enrollment.

	M	F	*p* Value
Sex *n* (%)	44 (62)	27 (38)	
Age (years, mean ± SD)	42.6 ± 8.6	40.4 ± 9.3	0.31
FTND (mean ± SD)	5.6 ± 2.3	5.1 ± 1.9	0.12
Packs/year (mean ± SD)	36.0 ± 14.3	26.5 ± 10.5	0.004
CPD (mean ± SD)	26.5 ± 7.9	22.3 ± 4.6	0.016

CPD: cigarettes per day; FTND: Fagerstrom Test for Nicotine Dependence.

### 3.2. Changes in Smoking Behaviour

**Figure 1:**
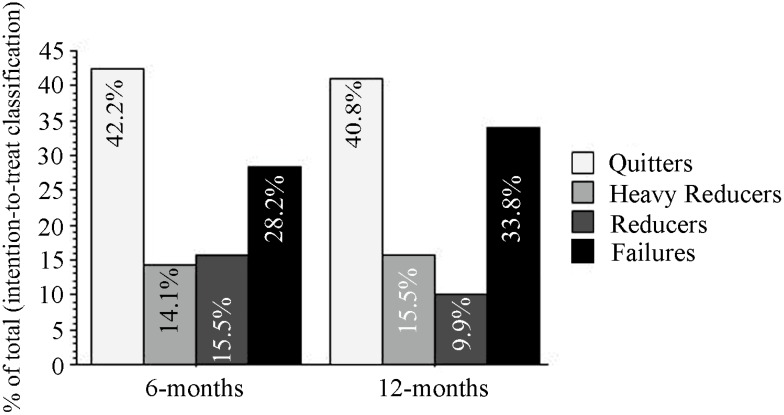
Distribution of smoking phenotype classification (intention-to-treat analysis) at 6 and 12 month follow-up visits.

Participants’ smoking status at baseline and at 6 and 12 month follow-up visits is presented in Figure 1. Taking the whole cohort of participants (*n* = 71), the cig/day use changed (mean and range) from 24.9 (15–50) at baseline to 4.0 (0–30) at 6 month and 2.6 (0–15) at 12 month (*p* < 0.0001). At 12 month, 29/71 subjects (40.8%) could be classified as quitters, 18/71 (25.4%) as reducers, of which 11 (15.5%) reduced their cig/day consumption by at least 80% from baseline, and 24/71 (33.8%) were classified as failures, of which 22 (31%) were lost to follow-ups.

Overall, combined smoking reduction and smoking abstinence was shown in 47/71 (66.2%) participants, with a mean (range) of 24.7 cig/day (15–50) at baseline, decreasing significantly to 2.2 cig/day (0–10) at 12 month (*p* < 0.0001), which is equivalent to an overall 89.1% reduction from baseline.

None of the individual characteristics (age, gender, pack/years, FTND) recorded at baseline were a significant predictor the smoking status at the final follow-up visit.

### 3.3. Changes in Products Choice

**Figure 2:**
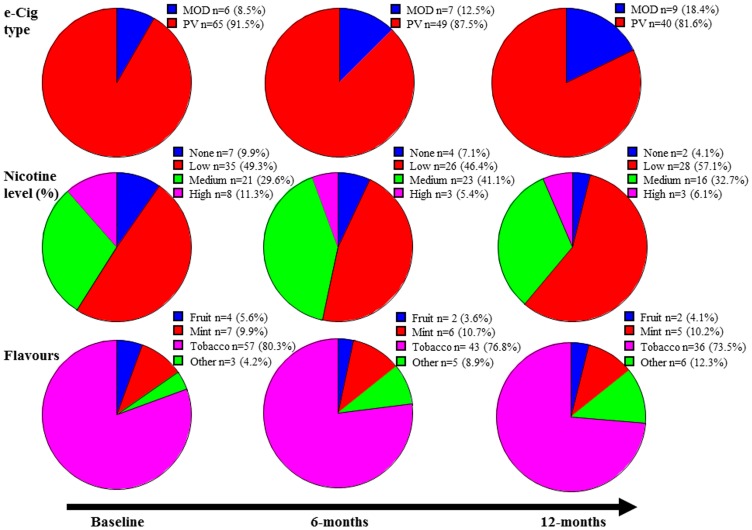
Details of e-Cigs type, e-liquid nicotine strengths (%) and flavours purchased at baseline and at 6 and 12 month follow-up visits. PV: personal vaporizers. MODs: more advanced devices. Low nicotine (4–9 mg/mL), medium nicotine (12–18 mg/mL), high nicotine (19–24 mg/mL).

Participants’ products choice at baseline and at 6 and 12 month follow-up visits is illustrated in Figure 2.

An increasing percentage of participants switched from standard refillable e-cigs (initial choice) to more advanced devices (MODs) during the study (from 8.5% at baseline to 18.4% at 12 month). Participants also tended to decrease the nicotine strength of their e-liquid with time. More users used a low (4–9 mg/mL) nicotine strength at 12 months, and, less users used a medium (12–18 mg/mL) nicotine strength at 12 month, compared to baseline. Some change did occur too for the preferred flavour used by the participants over time, but most of the participants in our study consistently preferred tobacco flavours over other flavours.

## 4. Discussion

E-cigs’ success rates have been reported in several clinical trials [10,11,15,16] and Internet surveys [18,19,20], but never in prospective studies under natural conditions. Here, we present results from the first prospective survey of changes in daily cigarette consumption in smokers making their first purchase at vape shops. The higher success rates observed in this study could reflect both a progress in the type of e-cigs used currently, and a better support and advice from the vape shop staff.

Success rates were not only high, but also stable thorough the whole observation period with quit rates of 42.2% in the intent-to-treat analysis at 6 month barely decreasing to 40.8% at 12 month. The reported quit rates are not only higher than those obtained with pharmaceutical products for the treatment of nicotine addiction [21,22], but also greater than those of first generation cigalikes [10,11]. In contrast, similar quit rates were observed in a recent prospective 6-month study with refillable e-cigs [15].

In addition to those quitting completely, 25.4% substantially reduced cigarette consumption. The prevalence of dual use (that is, use of both e-cigs and conventional cigarettes) in our survey is much lower than that reported for cigalikes [18,19,20]. Although dual use by leading to gradual reduction in cigarette consumption may aid future quit attempts [23,24], it is not known to what extent this behaviour may confer significant reduction in risk and reversal of harm in long-standing dual users.

The large number of consumers still using the product at 12 months (combined single and dual usage was 66.2%) and the high retention rate (69%) in this study may suggest that the products purchased were providing adequate satisfaction. This may be due to several factors including quality hardware, large selection of flavours and nicotine. Nicotine absorption using high quality e-vapour products has been shown to be consistently superior compared to cigalikes [25,26], which is compatible with a better suppression of the withdrawal symptoms. Last but not least, the high success rate in this study may be also attributable to participants self-selection (*i.e.*, smokers well motivated in trying e-cigs and making their first purchase at vape shops).

Nonetheless, about one third of smokers in this study failed to quit or to substantially reduce cigarette smoking with e-cigs. That reasons for failure were not collected in this study, but this could be due to the fact that probably not all smokers could find the adequate hardware-liquidware combination to allow a fulfilling vaping experience or that some unknown factor hindered their use under realistic conditions. It is not excluded also, that some of them may have persisted to use e-cigs, but went to buy their products in other vape shops than the one chosen for this study.

It is interesting that 69% of vape shop consumers went regularly back to their local vape shop for more personalized e-cig support and advice. This loyalty factor is perhaps a key informative finding and suggests that vape shop staff can promote healthier life-style changes in smokers.

As noted in other (internet) surveys, e-cig users tend to adapt their vaping experience over time [13,27]. This is reflected somewhat in the increased percentage of participants who switched from standard refillables (initial choice) to more advanced devices (MODs) in this study (from 8.5% at baseline to 18.4% at 12 month). Similarly, we observed a trend in decreasing the nicotine strength of their e-liquid, with more participants using low nicotine strength at 12 months compared to baseline, and inversely, with less participants using medium nicotine strength at 12 month compared to baseline. This could confirm that nicotine dependence decreases over time with e-cig use, as noted by other investigators [13,28], but cannot be validated in our study as we did not measure nicotine dependence at 12 month. The change in vaping experience was also the case for the preferred flavour used by the participants over time, although less significant in our study than in others [12,13,20], with the participants in our study consistently preferring tobacco flavours over any other flavour. This may reflect differences in study populations, vape shop consumers representing a more natural condition compared to those responding to online questionnaires.

There are some limitations in our study:

Firstly, this is a small prospective study (already stated in the text), hence the results observed may be due to bias and not due to a true effect; and consequently be interpreted with caution. However, despite being a small study we were able to detect positive significant changes for success outcomes.

Secondly, patients in this study may represent a self-selected sample, which is not representative of all smokers who switch to e-cigs.

Lastly, smoking abstinence was self-reported. However, self-reported number of cigarettes smoked per day in studies of this type is not subjected to the kind of biases observed in clinical trials where there is the tendency to claim abstinence [29].

This small uncontrolled study shows that combination of high quality e-vapour products together with personalized e-cig support and advice at vape shops promotes high success rates in smokers interested in trying this alternative to tobacco smoking. Complete tobacco cessation is the best outcome for smokers, but the powerful addictive qualities of smoked nicotine and of the ritualistic behavior of smoking create a huge hurdle, even for those with a strong desire to quit. Tobacco harm reduction (THR), the substitution of low-risk nicotine products for cigarette smoking, is a realistic strategy for smokers who have difficulty in quitting. E-cigs are the newest and most promising products for THR [30]. This approach has been recently exploited to reduce or reverse the burden of harm in smokers with mental health disorders and chronic airway diseases [31,32]. It is ironic, but the extent of displacement from tobacco smoking to regular vaping will also depend on how efficient e-cigs will become in replicating smokers’ smoking experience and how prevalent and helpful will be vape shops. As a matter of fact, substantial public health benefits (*i.e.*, increase in smoking cessation rates and a continued decline in smoking prevalence) are now reported in countries with high prevalence of vaping [33].

Improved products reliability and attractiveness might have contributed to the very low number of lost to follow-up and high success rates thus confirming the notion that these products are attractive substitutes for conventional cigarettes. Although larger longitudinal studies in vape shops are warranted to confirm these encouraging results, the notion that high quality e-vapour products together with personalized e-cig support and advice at vape shops can substantially decrease cigarette consumption, and allow a large number of smokers to quit should be taken into consideration by regulatory authorities seeking to adopt proportional measures for the vapour category [34].

## 5. Conclusions

Here we have shown for the first time that combining availability of appealing e-vapour products for smoking substitution with professional advice from vape shops staff it is possible to achieve high and stable success rates. By promoting healthier life-style changes in smokers, vape shops may become valuable allies in the fight against smoking. Larger studies are now needed to confirm these preliminary findings and to establish the importance of integrating these antismoking services into future tobacco control strategies.

## References

[1] Buchhalter A.R., Acosta M.C., Evans S.E., Breland A.B., Eissenberg T. (2005). Tobacco abstinence symptom suppression: The role played by the smoking-related stimuli that are delivered by denicotinized cigarettes. Addiction.

[2] Hughes J.R., Keely J., Naud S. (2004). Shape of the relapse curve and long-term abstinence among untreated smokers. Addiction.

[3] Polosa R., Benowitz N.L. (2011). Treatment of nicotine addiction: Present therapeutic options and pipeline developments. Trends Pharmacol. Sci..

[4] Stead L.F., Lancaster T. (2012). Combined pharmacotherapy and behavioural interventions for smoking cessation. Cochrane Database Syst. Rev..

[5] Alpert H.R., Connolly G.N., Biener L. (2013). A prospective cohort study challenging the effectiveness of population-based medical intervention for smoking cessation. Tob. Control.

[6] Pierce J.P., Cummins S.E., White M.M., Humphrey A., Messer K. (2012). Quitlines and nicotine replacement for smoking cessation: Do we need to change policy?. Annu. Rev. Public Health.

[7] Zhu S.H., Lee M., Zhuang Y.L., Gamst A., Wolfson T. (2012). Interventions to increase smoking cessation at the population level: How much progress has been made in the last two decades?. Tob. Control.

[8] Caponnetto P., Campagna D., Papale G., Russo C., Polosa R. (2012). The emerging phenomenon of electronic cigarettes. Expert Rev. Respir. Med..

[9] Caponnetto P., Russo C., Bruno C.M., Alamo A., Amaradio M.D., Polosa R. (2013). Electronic cigarette: A possible substitute for cigarette dependence. Monaldi Arch. Chest Dis..

[10] Caponnetto P., Campagna D., Cibella F., Morjaria J.B., Caruso M., Russo C., Polosa R. (2013). EffiCiency and Safety of an eLectronic cigAreTte (ECLAT) as tobacco cigarettes substitute: A prospective 12-month randomized control design study. PLoS One.

[11] Bullen C., Howe C., Laugesen M., McRobbie H., Parag V., Williman J., Walker N. (2013). Electronic cigarettes for smoking cessation: A randomised controlled trial. Lancet.

[12] Etter J.F., Bullen C. (2011). Electronic cigarette: Users profile, utilization, satisfaction and perceived efficacy. Addiction.

[13] Dawkins L., Turner J., Roberts A., Soar K. (2013). “Vaping” profiles and preferences: An online survey of electronic cigarette users. Addiction.

[14] Klein K.E. Health Markups on e-Cigarettes Turn Vacant Storefronts into “Vape Shops”. http://www.businessweek.com/articles/2013-10-03/healthymarkups-on-e-cigarettes-turn-vacant-storefronts-into-vape-shops.

[15] Polosa R., Caponnetto P., Maglia M., Morjaria J.B., Russo C. (2014). Success rates with nicotine personal vaporizers: A prospective 6-month pilot study of smokers not intending to quit. BMC Public Health.

[16] Adriaens K., van Gucht D., Declerck P., Baeyens F. (2014). Effectiveness of the electronic cigarette: An eight-week flemish study with six-month follow-up on smoking reduction, craving and experienced benefits and complaints. Int. J. Environ. Res. Public Health.

[17] Fagerstrom K.O., Schneider N.G. (1989). Measuring nicotine dependence: A review of the Fagerstrom Tolerance Questionnaire. J. Behav. Med..

[18] Siegel M.B., Tanwar K.L., Wood K.S. (2011). Electronic cigarettes as a smoking-cessation tool: Results from an online survey. Amer. J. Prev. Med..

[19] Etter J.F., Bullen C. (2014). A longitudinal study of electronic cigarette users. Addict. Behav..

[20] Farsalinos K.E., Romagna G., Tsiapras D., Kyrzopoulos S., Voudris V. (2014). Characteristics, perceived side effects and benefits of electronic cigarette use: A worldwide survey of more than 19,000 consumers. Int. J. Environ. Res. Public Health.

[21] Smith S.S., McCarthy D.E., Japuntich S.J., Christiansen B., Piper M.E., Jorenby D.E., Fraser D.L., Fiore M.C., Baker T.B., Jackson T.C. (2009). Comparative effectiveness of 5 smoking cessation pharmacotherapies in primary care clinics. Arch. Intern. Med..

[22] Polosa R., Caponnetto P. (2013). Advances in Smoking Cessation.

[23] Hughes J.R., Carpenter M.J. (2005). The feasibility of smoking reduction: An update. Addiction.

[24] Walker N., Bullen C., McRobbie H. (2009). Reduced-nicotine content cigarettes: Is there potential to aid smoking cessation?. Nicotine Tob. Res..

[25] Dawkins L., Corcoran O. (2014). Acute electronic cigarette use: Nicotine delivery and subjective effects in regular users. Psychopharmacology.

[26] Farsalinos K.E., Spyrou A., Tsimopoulou K., Stefopoulos C., Romagna G., Voudris V. (2014). Nicotine absorption from electronic cigarette use: Comparison between first and new-generation devices. Sci. Rep..

[27] Farsalinos K.E., Romagna G., Tsiapras D., Kyrzopoulos S., Spyrou A., Voudris V. (2013). Impact of flavour variability on electronic cigarette use experience: An internet survey. Int. J. Environ. Res. Public Health.

[28] Farsalinos K.E., Romagna G., Tsiapras D., Kyrzopoulos S., Voudris V. (2013). Evaluating nicotine levels selection and patterns of electronic cigarette use in a group of “vapers” who had achieved complete substitution of smoking. Subst. Abuse.

[29] Wong S.L., Shields M., Leatherdale S., Malaison E., Hammond D. (2012). Assessment of validity of self-reported smoking status. Health Rep..

[30] Polosa R., Rodu B., Caponnetto P., Maglia M., Raciti C. (2013). A fresh look at tobacco harm reduction: The case for the electronic cigarette. Harm Reduct. J..

[31] Caponnetto P., Auditore R., Russo C., Cappello G.C., Polosa R. (2013). Impact of an electronic cigarette on smoking reduction and cessation in schizophrenic smokers: A prospective 12-month pilot study. Int. J. Environ. Res. Public Health.

[32] Polosa R., Morjaria J.B., Caponnetto P., Caruso M., Strano S., Battaglia E., Russo C. (2014). Effect of smoking abstinence and reduction in asthmatic smokers switching to electronic cigarettes: Evidence for harm reversal. Int. J. Environ. Res. Public Health.

[33] West R., Brown J., Beard E. Smoking Toolkit Study. Trends in Electronic Cigarette Use in England. http://www.smokinginengland.info/latest-statistics/.

[34] Saitta D., Ferro G.A., Polosa R. (2014). Achieving appropriate regulations for electronic cigarettes. Ther. Adv. Chronic Dis..

